# Causal Relationships Between Environmental Exposures, Iron Metabolism, Hematuria Markers, and Rheumatoid Arthritis: An Investigation Using Mendelian Randomization

**DOI:** 10.3390/biomedicines13020513

**Published:** 2025-02-19

**Authors:** Chao Wang, Wenqing Xie, Chenggong Wang, Yong Zhu, Da Zhong

**Affiliations:** 1 Department of Orthopedics, Xiangya Hospital, Central South University, Changsha 410008, China; chaowang@csu.edu.cn (C.W.); xiewenqing@csu.edu.cn (W.X.); wangchenggong@csu.edu.cn (C.W.); 2 National Clinical Research Center for Geriatric Disorders, Xiangya Hospital, Central South University, Changsha 410008, China

**Keywords:** rheumatoid arthritis, iron homeostasis, hematuria markers, Mendelian randomization, genome-wide association studies

## Abstract

**Background:** Rheumatoid arthritis (RA) is a globally prevalent chronic inflammatory disease. Environmental exposures, such as air pollution and smoking, are considered potential risk factors. However, the causal relationships and underlying mechanisms between these factors and RA are not fully understood. **Methods:** This study utilized large-scale genome-wide association studies (GWASs) from European ethnic backgrounds and employed bidirectional two-sample Mendelian randomization (MR) to investigate the relationships between air pollution, smoking, and RA. Genetic correlations were assessed using linkage disequilibrium score regression (LDSC). Furthermore, mediation analysis was conducted to evaluate the potential mediating roles of iron metabolism and urinary biomarkers in these relationships. **Results:** The MR analysis revealed that genetically predicted lifetime smoking intensity was associated with an 85% increased risk of RA. Subgroup analysis differentiating between seropositive RA (SPRA) and seronegative RA (SNRA) showed a causal association with SPRA, but not with SNRA. C-reactive protein was identified as a mediator in the relationship between lifetime smoking and both RA and SPRA, mediating 18.23% and 32.45% of the effects, respectively. Genetic correlation analysis further confirmed a positive genetic association between smoking and both RA and SPRA. **Conclusions:** This study provides significant insights into the genetic and causal connections between air pollution, smoking, and the development of RA, highlighting the mediating role of C-reactive protein. These findings not only offer new perspectives on how smoking might enhance RA risk through inflammatory pathways but also underscore the importance of reducing smoking exposure in public health strategies.

## 1. Introduction

Rheumatoid arthritis (RA) is an immune-mediated systemic inflammatory disorder characterized by synovial joint damage in the hands and feet, along with extra-articular manifestations, linked to progressive disability, premature mortality, and substantial socio-economic costs [1,2,3]. Globally, RA affects up to 1% of the adult population, with an incidence that appears to be increasing annually [4,5]. Studies indicate that both the age-standardized prevalence and disability-adjusted life years increase with age, with higher rates observed in females. Incidence peaks in females and males occur in the 70–74 and 75–79 age groups, respectively [6]. As global populations age, projections suggest an 80.2% increase in RA cases from 2020 to 2050, representing a significant societal burden, especially in low- and middle-income countries [7,8].

The precise etiology of RA involves a complex interplay among environmental factors, genetic predisposition, and immune dysregulation, with genetics accounting for nearly 60% of RA risk [9,10,11]. Air pollution, particularly exposure to particulate matter (PM 2.5 μm, PM 10 μm) and nitrogen oxides (NO_2_, NOx), has been linked to systemic inflammation and various diseases, including RA [12,13]. Studies have shown mixed results: while some indicate an increased RA risk with exposure to traffic-related pollutants [14,15], others found no significant associations [16]. Current research stratifies RA phenotypes based on serological antibodies—rheumatoid factor (RF) or ACPA—into seropositive RA (SPRA, positive for RF and/or ACPA) or seronegative RA (SNRA, negative for both RF and ACPA), each typically associated with distinct genetic susceptibilities and immunopathogenic pathways [17,18]. Despite these distinctions, therapeutic guidelines often do not differentiate between SNRA and SPRA, potentially overlooking nuances in treatment needs, as highlighted in recent studies [19]. Additionally, addressing the unmet needs in RA treatment, particularly the efficient delivery of pharmacological agents to target sites, nanomedicine emerges as a promising field. Nanotechnological approaches in RA, such as the use of targeted nanoparticle delivery systems, offer the potential to enhance treatment efficacy and are currently under active research [20].

RA progresses through two main phases: the pre-RA phase and the RA phase [17]. The pre-RA phase involves systemic autoimmunity with biomarkers such as anti-CCP and RF, which are detectable years before symptoms appear. This phase is critical for SPRA, as these biomarkers help in early diagnosis and indicate a severe disease trajectory. SNRA lacks these markers, leading to diagnostic challenges and potentially milder yet unpredictable progression. In the RA phase, where symptoms are evident, biomarkers in SPRA aid in diagnosis and treatment planning. In contrast, their absence in SNRA complicates management, affecting treatment timing and selection. Recognizing these phase distinctions is key to devising effective, personalized treatment strategies for RA. Despite extensive research, the role of environmental factors such as smoking and air pollution in RA remains partially understood, underscoring the need for high-quality research designs to validate these causal relationships. Although randomized controlled trials are the gold standard for causal inference, ethical constraints or the high costs associated with such trials limit their applicability. Observational studies investigating the links between environmental exposures and RA often rely on cross-sectional or case-control designs, which may not adequately adjust for confounding factors within the population and fail to establish the directionality of causal relationships. Thus, there remains a need for more robust research designs to effectively verify causal links.

Advances in genetic epidemiology have introduced MR as a potent tool to assess causality within complex biological systems. MR offers a higher level of evidence compared to case-control and cohort studies by utilizing genetic variants as instrumental variables (IVs) [21]. These alleles are randomly assorted during meiosis and are generally unaffected by environmental factors or the disease process, thus enabling estimation of the impact of exposures on outcomes while significantly minimizing confounding, strengthening the exposure–outcome association, and avoiding reverse causation. LDSC represents another classical tool for genetic correlation analysis. As a robust method derived from GWAS summary statistics to estimate heritability and genetic correlation, LDSC can help differentiate true polygenicity from confounding biases such as population stratification and cryptic relatedness [22].

RA is a complex disease involving multiple biological pathways, where iron metabolism and various blood and urinary biomarkers play pivotal roles. Indicators of iron metabolism, such as serum iron, total iron-binding capacity, transferrin saturation, and ferritin, not only reflect the state of iron metabolism but are also closely linked with immune function and inflammation. During inflammatory responses, levels of these markers often change, and their aberrant expression in the autoimmune disease RA is associated with the severity and progression of the disease [23,24]. Additionally, ferroptosis induced by iron metabolic dysregulation in lesions has been reported to be connected with the onset and progression of inflammatory arthritis [25]. Other markers in blood and urine, such as C-reactive protein and HDL cholesterol, are also used to assess disease activity and response to treatment in RA patients [26,27], further underlining the importance of choosing these biomarkers for study. Within the genetic context of RA, mediation MR allows for a rigorous exploration of how biomarkers mediate the direct and indirect impacts leading to RA. Based on their common use in diagnostics and chronic disease assessment, we attempt to unveil their potential in linking environmental exposure to the development of RA.

This study aims to integrate various genetic methodologies to investigate the genetic associations between environmental exposures and RA, delving deeper into the potential mechanisms and mediating pathways involved. Employing a forward MR approach, complemented by sensitivity analyses and LDSC for robust validation, we estimated the causal impact of environmental exposure on RA. Further mediation MR and enrichment analyses explored the potential mechanisms by which environmental exposures might influence RA. Our findings indicate that lifetime smoking significantly increases the risk of RA, particularly SPRA rather than SNRA. Additionally, this paper reveals for the first time the mediating role of CRP in the link between lifetime smoking and both RA and SPRA, elucidating the potential mechanism by which smoking exacerbates RA through inflammatory pathways. These insights not only clarify the genetic and molecular pathways through which smoking aggravates RA but also underscore the urgent need to reduce smoking exposure in public health initiatives and offer potential strategies for the prevention and treatment of RA.

## 2. Materials and Methods

### 2.1. Study Design

**Figure 1:**
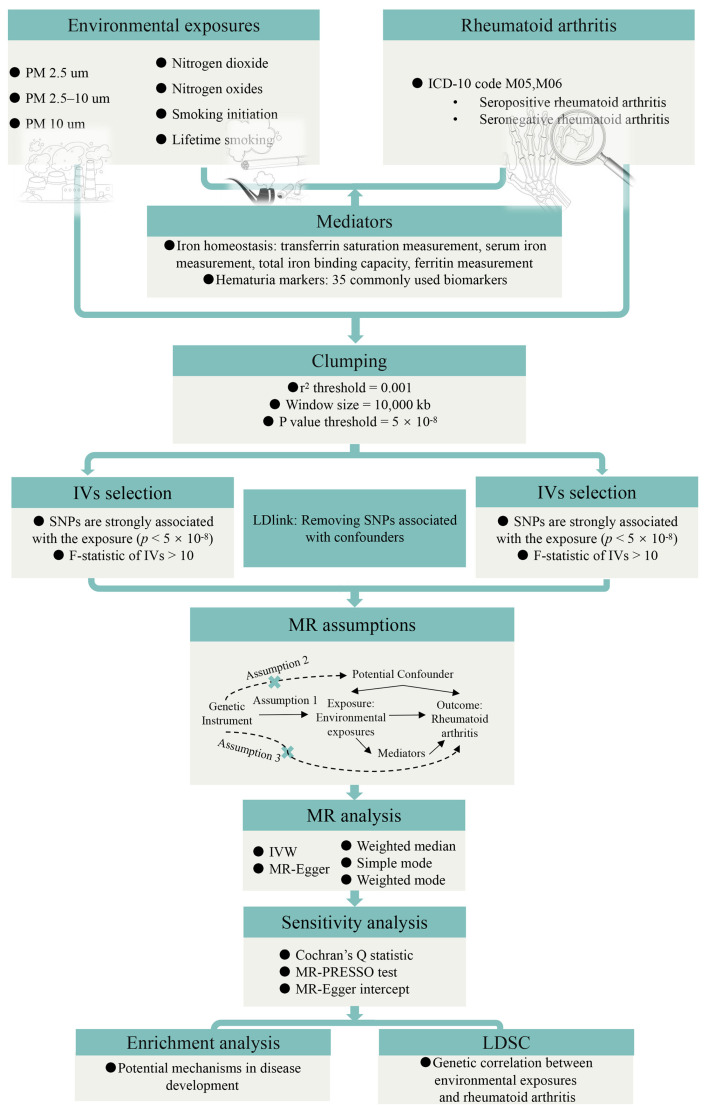
Workflow of this study. Assumption 1: The instrumental variables should have a strong correlation with the exposures. Assumption 2: The instrumental variables must be unrelated to any potential confounders that could influence the relationship between the exposure and the outcome. Assumption 3: The instrumental variables should not have a direct effect on the outcomes.

The workflow of our study is depicted in Figure 1. Our investigation was conducted employing a bidirectional two-sample MR approach, and our MR analysis was crafted in accordance with the STROBE-MR guidelines (Supplementary STROBE checklist). Initially, we implemented MR to examine the causal relationship between environmental exposures and RA. To better understand this connection, RA was categorized into seropositive and seronegative subtypes. For positive findings, further scrutiny was applied to their association with diverse RA subtypes. Thereafter, sensitivity analyses were undertaken to ascertain the robustness of our discoveries and to explore potential sources of heterogeneity. Subsequently, mediation MR analysis examined the mediating role of iron metabolism and hematuria markers in the linkage between environmental exposures and RA. This analysis allowed us to quantify the proportion of effects mediated by these factors, further illuminating underlying mechanisms, in tandem with enrichment analyses. Ultimately, we employed LDSC to probe potential genetic correlations between environmental exposures and RA, despite the possibility of non-causal relationships.

### 2.2. Data Sources

Our research utilized data accessible from public databases; participants in the GWASs were of European descent.

The environmental exposures investigated predominantly encompass air pollution and smoking. Summary statistics on the association between air pollution and RA were drawn from the UK Biobank—a large-scale prospective study involving over half a million UK participants whose phenotypic details, genetic nuances, and complete genomic genotyping data have been published (http://www.ukbiobank.ac.uk, accessed on 1 April 2024.) [28,29]. Five GWAS datasets relating to air pollution were selected, including PM 2.5 μm (*n* = 423,796), PM 2.5–10 μm (*n* = 423,796), PM 10 μm (*n* = 455,314), Nitrogen dioxide (*n* = 456,380), and Nitrogen oxides (*n* = 456,380). Air pollution indices were measured using land-use regression (LUR) models [30]. Smoking was divided into smoking initiation (*n* = 341,427) and lifetime smoking (*n* = 462,690), confirming the heritability of these phenotypes/behaviors as reported by the Tobacco and Genetics Consortium. Specifically, data on smoking initiation came from the GSCAN meta-genome-wide association study summary statistics, which defined it as a binary variable comparing never smokers to smokers who smoked over 100 cigarettes in their lifetime [31]. The lifetime smoking index derived from the UK Biobank factored various aspects of participants’ smoking habits, including the severity and duration of smoking, onset and cessation, eventually combined with an estimated half-life constant reflecting exponential decline impacts on health outcomes posed at specific times—ultimately serving as a multifaceted evaluative measure for smoking [32].

RA (ICD-10 code M05, M06) data were derived from the FinnGen Consortium’s GWAS summary dataset available online (https://r9.finngen.fi, accessed on 1 April 2024.) [33], encompassing 12,555 RA cases and 240,862 controls. It detailed data for seropositive rheumatoid arthritis (SPRA, *n* = 377,272) as well as seronegative rheumatoid arthritis (SNRA, *n* = 288,912), allowing for more thorough validation.

Iron metabolism data originated from recent GWAS meta-analyses incorporating four key iron homeostasis biomarkers: transferrin saturation (*n* = 131,471), serum iron content (*n* = 163,511), total iron-binding capacity (*n* = 135,430), and ferritin (*n* = 246,139) [34]. Data pertaining to blood and urine biomarkers were also accessible online (https://gwas.mrcieu.ac.uk/, accessed on 1 April 2024.), featuring comprehensive statistics for 35 common biomarkers spanning GWAS IDs from GCST90001391 to GCST90002121 [35].

### 2.3. Genetic Instruments Selection

The selection of Single Nucleotide Polymorphisms (SNPs) for our study was conducted via a systematic and stringent process delineated as follows: (1) To ensure robust statistical significance, a genome-wide significance threshold of *p* ≤ 5 × 10^−8^ was employed. This threshold is conventionally adopted in GWAS to mitigate the possibility of false positives arising from multiple testing. (2) SNPs displaying a minor allele frequency below 0.05 were meticulously excluded from our analyses to maintain reliable genetic variance. (3) SNPs harboring mismatched allele pairs, such as C/T and C/A, were excluded from analysis. (4) Palindromic variants with ambiguous strands, such as C/G or A/T, were also eliminated from our dataset, a precaution that is vital for avoiding strand ambiguity, which could introduce errors during analysis. (5) The genetic variants that successfully passed the aforementioned filters underwent additional processing in Plink. This software facilitated clustering using specific parameters to identify lead SNPs with settings configured at a window size of 10,000 kb and an r2 less than 0.001, using LDlink to eliminate all potential confounding SNPs (https://ldlink.nih.gov/?tab=ldtrait, accessed on 1 April 2024.) [36].

### 2.4. MR Analysis

A bidirectional MR approach was implemented to rigorously investigate the causal relationships between environmental exposures and RA on a genetic scale. SNPs with low F-statistics (<10) were excluded from our analysis to dodge significant weak instrument bias. The Wald ratio (WR) method was utilized when only a single SNP was available, whereas the inverse-variance weighted (IVW) method served as the primary methodology for estimating causal relationships between exposure and outcome when two or more SNPs were available. Sensitivity analyses were enriched by employing four additional MR methods, including MR-Egger regression, simple mode, weighted median, and weighted mode. Heterogeneity in the IVW estimates was assessed using Cochran’s Q test. The MR Egger intercept was evaluated to ascertain pleiotropy. The Mendelian randomization Pleiotropy RESidual Sum and Outlier (MR-PRESSO) framework, which encompasses global and outlier tests, was deployed to detect and rectify pleiotropy and potential outliers. After the exclusion of pertinent outliers, causal estimates were recalculated, cementing a robust causal inference that considered potential biases and confounding factors inherent in the data. Further two-step MR analyses were pursued to explore whether iron metabolism and hematuria markers mediate the causal relationship between exposure and outcome.

### 2.5. Enrichment Analysis

In our investigation, we commenced by meticulously selecting SNPs from genomic data that exhibited significant correlations with environmental exposures and the risk and phenotypes associated with RA. These SNPs were cataloged and annotated using the public database dbSNP (https://www.ncbi.nlm.nih.gov/snp/, accessed on 1 April 2024.). Subsequently, we undertook a comprehensive enrichment analysis of the functions and pathways of the corresponding genes, endeavoring to uncover their potential roles and biological pathways in disease mechanisms. Specifically, using Metascape (https://metascape.org, accessed on 1 April 2024.) [37], we conducted analyses related to Gene Ontology (GO) and the Kyoto Encyclopedia of Genes and Genomes (KEGG), encompassing biological processes (BPs), molecular functions (MFs), cellular components (CCs), Reactome Gene Sets, Canonical Pathways, CORUM, and WikiPathways. This integrative approach employing both GO and KEGG not only augmented our systematic understanding of gene interactions but also facilitated the extraction of crucial biological insights from the intricate genetic milieu by elucidating the associations between gene functions and pathways.

### 2.6. LDSC

LDSC serves as a pivotal methodology utilizing GWAS data to estimate the genetic correlation between two traits. This method proves particularly invaluable in contexts characterized by high polygenicity, enabling us to probe the genetic overlap between environmental exposures and RA. Genetic correlations (rg) are estimated using LDSC software (https://github.com/bulik/ldsc, accessed on 1 April 2024.), employing cross-trait Linkage Disequilibrium Score Regression [22].

### 2.7. Statistical Analysis

R software, version 4.3.1, was employed to conduct MR and other analytical methods, utilizing the “TwoSampleMR” package. The results are expressed in terms of odds ratios (ORs), along with their 95% confidence intervals (CIs) per standard deviation increment. The threshold of statistical significance was defined as *p* < 0.05. The proportional mediation was derived based on the formula in which β represents the total effect obtained from preliminary analyses, whereas β1 and β2 denote the impact of exposure characteristics on mediator factors and the impact of said mediator factors on outcomes, respectively: proportion = (β1 × β2)/β.

## 3. Results

### 3.1. Causal Relationships Between Environmental Exposures and RA

#### 3.1.1. Influence of Environmental Exposures on RA Through Discovery MR

Adhering to the criteria for the selection of IVs, we carefully selected 7, 144, 57, 22, 355, 119, and 85 SNPs significantly associated with smoking initiation, lifetime smoking, PM 2.5 μm, PM 2.5–10 μm, PM 10 μm, nitrogen dioxide, and nitrogen oxides, respectively, across the genome.

**Figure 2:**
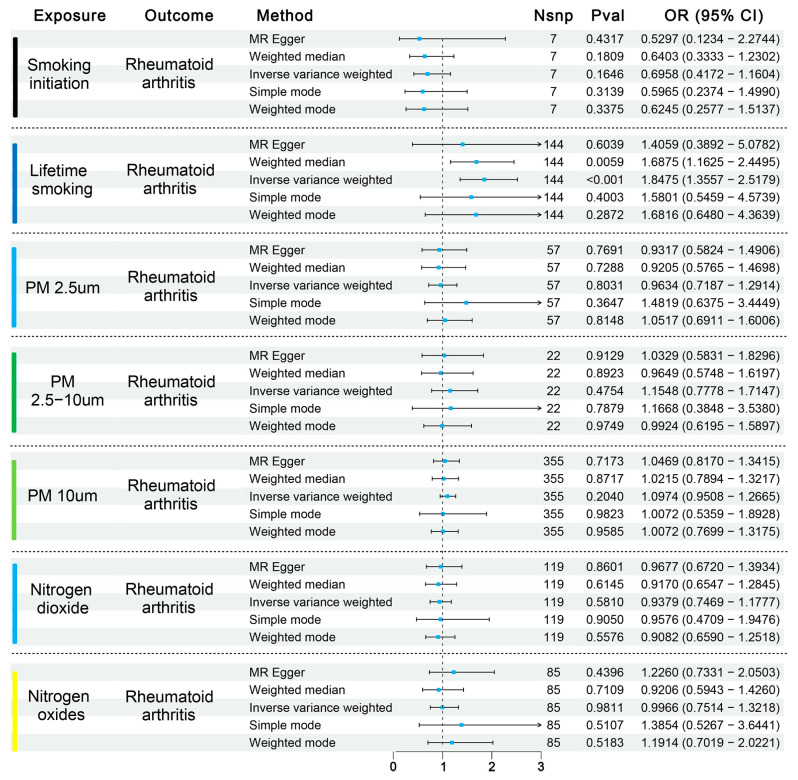
Effects of environment exposures on rheumatoid arthritis. Results from MR analyses showing the potential casual effects of environment exposures (smoking initiation, lifetime smoking, PM 2.5 μm, PM 2.5–10 μm, PM 10 μm, nitrogen oxides, and nitrogen dioxide) on rheumatoid arthritis.

All exposures were analyzed using the inverse-variance weighted (IVW) model as the principal method in our MR analyses. The primary results are presented in Figure 2. Among the environmental exposures assessed, the IVW approach revealed that genetically determined levels of lifetime smoking (per 1-SD increase) were associated with an 85% increase in the odds of developing RA (OR = 1.85; 95% CI: 1.36–2.52; *p* = 1.02 × 10^−4^). This finding was consistent in the weighted median model analysis (OR = 0.71; *p* = 0.01). The remaining three MR analyses did not detect statistically significant associations but demonstrated similar trends of variation. Further sensitivity analysis for lifetime smoking is documented in Supplementary Table S1. MR-Egger regression intercept analysis indicated no significant pleiotropy. Cochran’s IVW Q-test suggested potential heterogeneity among instrumental variables. Subsequent Mendelian randomization pleiotropy RESidual sum and outlier (MR-PRESSO) analysis identified outliers (rs12481282, rs62155874, rs732083), and upon correction for these outliers, the association remained significant (PRESSO outliers corrected OR = 2.06; *p* = 2.18 × 10^−6^). Screening of SNPs by linkage disequilibrium traits confirmed no direct connections between SNPs and outcomes, nor any associations with potential confounders affecting exposure and outcome relationships.

#### 3.1.2. Influence of Smoking on SPRA or SNRA Through Confirmatory MR

**Figure 3:**
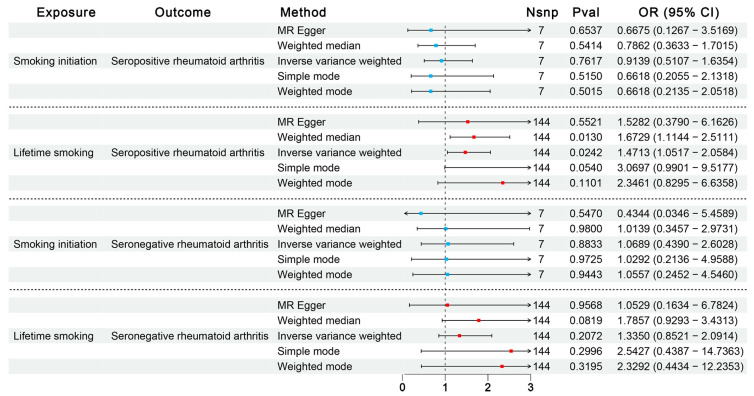
Effects of smoking on subtypes of rheumatoid arthritis. Results from MR analyses, showing the potential casual effects of smoking (smoking initiation, lifetime smoking) on seropositive and seronegative rheumatoid arthritis.

Previous steps unveiled a causal link between smoking and RA. To bolster confidence in our MR study findings, we utilized data from the FinnGen consortium subdividing RA into SPRA and SNRA and carried out confirmatory MR analyses, as shown in Figure 3. Intriguingly, post-subdivision analyses highlighted a robust association between genetically predicted lifetime smoking and SPRA (IVW OR = 1.47; 95% CI = 1.05–2.06), but found no causal relationship with SNRA. No significant effects of smoking initiation on either SPRA or SNRA were observed. Subsequent outlier detection during sensitivity analyses (rs12481282, rs77653640) affirmed a close association with SPRA after outlier corrections (PRESSO outliers corrected OR = 1.60; *p* = 3.40 × 10^−3^).

#### 3.1.3. Influence of RA on Environmental Exposures

In order to evaluate the potential for reverse causation, RA, SPRA, and SNRA were examined as exposures, with environmental exposures assessed as outcomes through MR analysis. The results indicated that the genetic susceptibilities to RA, SPRA, and SNRA had no influence on any environmental exposures, as depicted in Supplementary Table S2.

### 3.2. Two-Step MR Analysis

#### 3.2.1. Causal Effects of Lifetime Smoking on Mediator Factors

**Figure 4:**
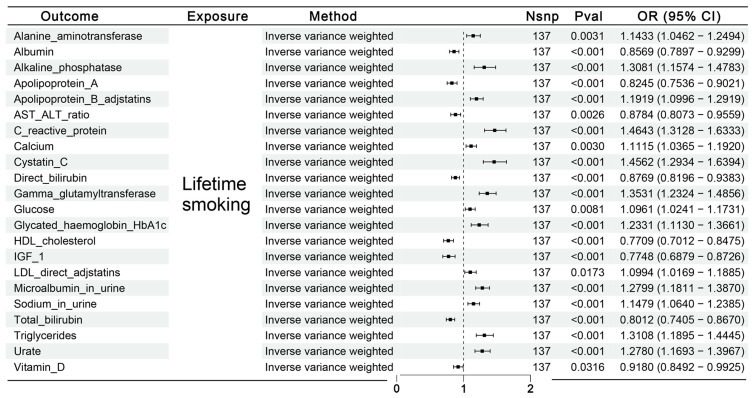
Effects of lifetime smoking on iron metabolism and hematuria markers. Results from first-step MR analyses show the potential causal relationship between genetically predicted lifetime smoking and iron metabolism and hematuria markers, using IVW methods.

Supplementary Table S3 elucidates the causal impact of lifetime smoking on iron metabolism and hematuria markers. Employing the IVW method, a putative causal relationship was observed between lifetime smoking and 22 urinary biomarkers; however, no correlations were observed with iron metabolism markers. These potential mediators were subsequently utilized for further MR analyses, illustrated in Figure 4.

#### 3.2.2. Causal Effects of Possible Mediator Factors on RA or SPRA

Further estimates elucidated the effects of these 22 potential hematuria markers related to lifetime smoking on the risk of RA or SPRA, as documented in Supplementary Table S4. Notable correlations included positive associations with RA, such as C-reactive protein (OR = 1.34, 95% CI: 1.07–1.69) and Cystatin C (OR = 1.11, 95% CI: 1.01–1.23). In contrast, albumin (OR = 0.84, 95% CI: 0.71–0.98) and glucose (OR = 0.80, 95% CI: 0.65–0.97) levels were negatively associated with RA risk. Similar trends consistent with RA were also observed for SPRA, concerning biomarkers such as C-reactive protein, albumin, and glucose.

#### 3.2.3. Mediation Proportion

**Figure 5:**
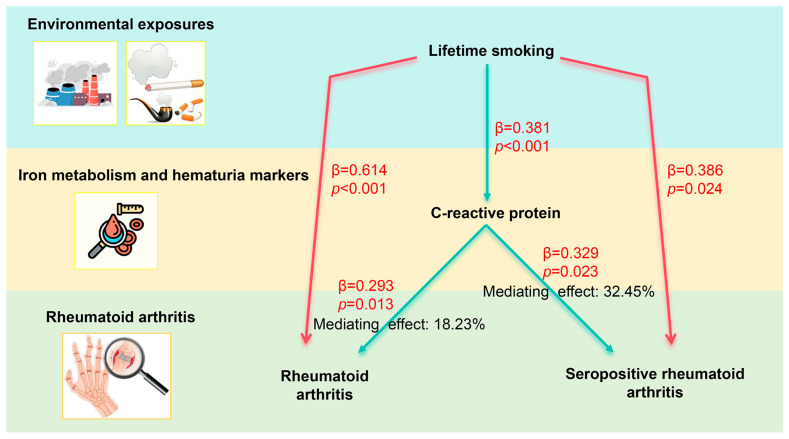
Mendelian randomization analyses show causal effects of iron metabolism and hematuria markers on environmental exposures and rheumatoid arthritis. The diagram displays the mediation mode of “lifetime smoking-C reactive protein-rheumatoid arthritis or seropositive rheumatoid arthritis” in two-step Mendelian randomization. Beta values (β) indicate the causal effect estimates using the inverse-variance weighted method (truncated at *p* < 0.05). Characters colored in red signify positive associations.

**Figure 6:**
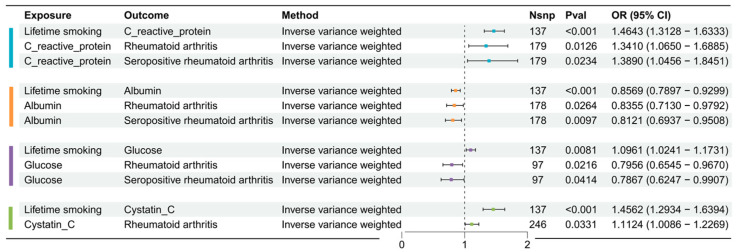
Two-step Mendelian randomization.

Based on the MR outcomes, four and three potential hematuria markers involved in mediating the effect of lifetime smoking on RA and SPRA, respectively, were identified, along with calculated mediation proportions and 95% Cis, detailed in Supplementary Table S5. Ultimately, we confirmed C-reactive protein as a definitive mediator in the association between lifetime smoking and both RA (mediation proportion: 18.23%; 95% CI: 5.29–31.16%) and SPRA (mediation proportion: 32.45%; 95% CI: 5.99–58.9%) (Figure 5). Despite the non-significant results after the calculations, the remaining biomarkers successfully passed the two-step MR validation and are still considered potential mediators (Figure 6).

### 3.3. Results of Enrichment Analysis

**Figure 7:**
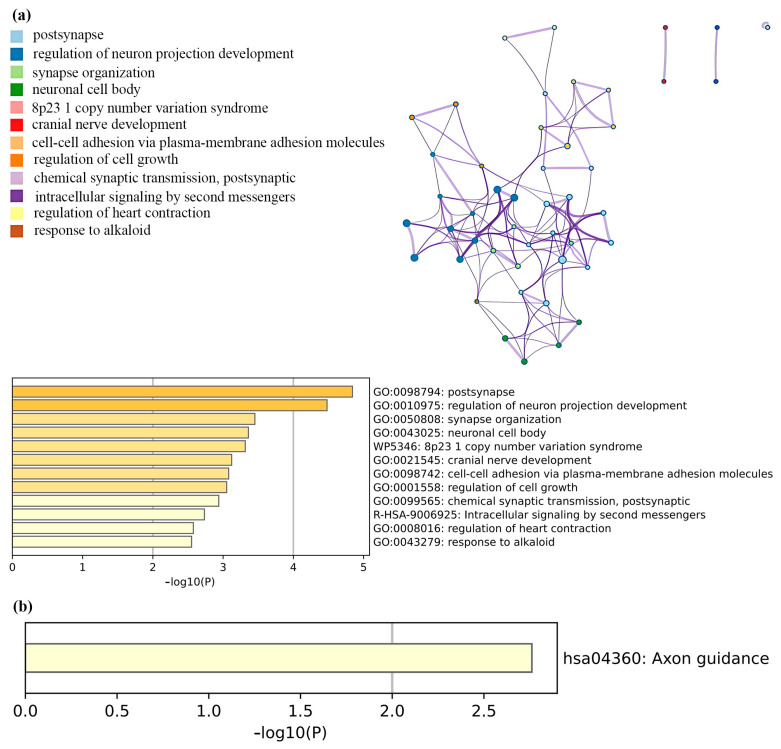
Enrichment analysis. (**a**) Network of enriched terms: colored by cluster ID, where nodes that share the same cluster ID are typically close to each other; bar graph of enriched terms across gene lists, colored by *p*-values. (**b**) KEGG pathway enrichment analysis.

Upon transforming the SNPs significantly associated with smoking and RA into genes, enrichment analysis was conducted, detailed in Figure 7 and Supplementary Table S6. The top five clusters included postsynapse, regulation of neuron projection development, synapse organization, neuronal cell body, and the 8p23.1 copy number variation syndrome (Supplementary Table S7). Moreover, the results from KEGG pathway enrichment analysis suggested that the genetic influence of lifetime smoking on RA is predominantly concentrated in the axon guidance pathway.

### 3.4. Genetic Correlation

Lastly, GWAS summary data for environmental exposures, RA, and SPRA were utilized for a genetic correlation analysis. As illustrated in Table 1, consistent with MR study outcomes, there exists a significant positive genetic correlation between lifetime smoking and RA (correlation coefficient = 0.158, *p* = 1.22 × 10^−5^) and SPRA (correlation coefficient = 0.104, *p* = 5.81 × 10^−3^). Notably, despite the absence of a meaningful causal link revealed in the MR analyses, linkage disequilibrium score regression (LDSC) analysis identified a significant positive genetic correlation between smoking initiation and both RA or SPRA.

## 4. Discussion

This study utilized MR to investigate the potential genetic connections and causal relationships between environmental exposures and RA. RA, with its rising incidence and disability rates, poses significant challenges to individuals, health systems, and society at large. The etiology of RA is not completely understood, but is thought to involve a combination of genetic susceptibility, environmental factors, and immune system dysfunction [38]. Epidemiological studies and clinical observations frequently highlight environmental factors, particularly smoking, as key risk factors that exacerbate the disease [39,40]. Moreover, smoking is believed to aggravate joint damage and bone erosion by impairing immune system functions [41,42]. Exploring the genetic and causal associations between smoking and RA not only helps reveal the disease’s underlying mechanisms but could also inform future strategies for prevention and treatment. By analyzing large-scale GWAS data from European populations, we discovered that lifelong smoking significantly elevates the risk of developing RA, especially SPRA. Additionally, C-reactive protein (CRP), as an inflammatory biomarker, appears to mediate the relationship between smoking and both RA and SPRA. This indicates that inflammatory pathways may be key mechanisms by which smoking influences RA progression. These insights provide a new perspective on how smoking exacerbates RA through genetic and molecular pathways and highlight the critical role of reducing smoking exposure in public health strategies.

Our results show that smoking is not only associated with a generalized risk for RA but is particularly linked with the SPRA phenotype. The confidence interval for the odds ratio (95% CI: 1.36–2.52) suggests a strong and robust association between lifetime smoking and RA. This interval indicates that the observed effect is highly unlikely to be due to chance, reinforcing the reliability of our findings. These results align with previous observational studies that frequently reported a connection between smoking and heightened RA risk [43]. However, by utilizing genetic instrumental variables, this study mitigates potential confounders and reverse causality, thus providing stronger evidence for smoking as a risk factor for RA. The positive genetic correlation supports the hypothesis that interventions aimed at reducing smoking behavior could potentially lower the risk of developing RA. Conversely, the lack of a significant genetic correlation between air pollution and RA in our findings suggests independent genetic pathways. This implies that while both air pollution and RA might involve inflammatory processes, the genetic determinants that predispose individuals to RA do not significantly overlap with those that increase susceptibility to air pollution. This distinction is crucial for understanding the specific genetic contributions to RA and can help tailor public health interventions that address different environmental risk factors independently. Research, including a cohort study of Swedish women, highlights that the intensity and duration of smoking correlate with RA risk, persisting up to 15 years after cessation [44]. Further, findings from the Nurses’ Health Study suggest that the risk associated with smoking decreases over time post-cessation, with long-term quitters showing reduced risks, particularly for SPRA. However, this risk remains slightly elevated even decades after quitting [45]. Other studies have indicated varying impacts of smoking on different RA serologic phenotypes, with a notable susceptibility to SPRA in individuals with certain genetic markers [46]. Beyond its role as a risk factor, smoking adversely affects RA disease activity and treatment outcomes. Smokers exhibit higher disease activity and reduced remission rates during treatment compared to non-smokers, and these effects extend to elevations in specific autoantibodies and cardiovascular risks [47,48,49,50]. Smoking also independently predicts worse radiographic progression in early RA [51].

In our study, mediation analysis was employed to explore the potential biological pathways through which lifetime smoking may influence the development of RA. Notably, CRP, a well-established biomarker of inflammation, was identified as a significant mediator in this relationship. Our analysis revealed that CRP mediates 18.23% of the effect of lifetime smoking on RA. This substantial proportion suggests that inflammatory pathways, potentially triggered or exacerbated by smoking, play a critical role in the pathogenesis of RA. The significance of identifying CRP as a mediator is twofold. Firstly, it provides a quantifiable measure of how much of the smoking–RA relationship is attributable to inflammation. This is crucial because inflammation is a central feature of RA, and understanding its drivers can help target therapeutic strategies more effectively. Secondly, the mediation by CRP underscores the biological plausibility of smoking as a risk factor for RA, reinforcing the need for interventions aimed at reducing smoking rates as a preventive measure against RA. Furthermore, the mediation analysis not only highlights the importance of inflammatory pathways but also provides a clearer picture of the underlying mechanisms by which environmental factors such as smoking can lead to chronic diseases, including RA. This insight is valuable for both clinicians and public health professionals in managing and preventing RA. A longitudinal study involving 6028 participants demonstrated that persistent smokers had elevated levels of CRP, indicating an association between smoking and chronic low-grade inflammation [52]. Our analysis suggests that CRP mediates a significant portion of the risk of RA, especially of SPRA, attributable to smoking, shedding light on potential pathological mechanisms. Specifically, the oxidative stress and free radicals generated by smoking initiate an inflammatory response in the body, marked by an increase in CRP levels. CRP not only participates in activating inflammatory mediators but may also enhance the interaction between leukocytes and endothelial cells by promoting the expression of cell adhesion molecules. This facilitates the migration and infiltration of inflammatory cells into joint tissues [53]. Furthermore, CRP can activate the complement system, intensifying the inflammatory response, which plays a crucial role in the joint inflammation and tissue destruction seen in RA [54]. Additionally, elevated CRP levels are linked to functional changes in immune cells, such as enhanced macrophage phagocytic activity and a shift in T cells towards a Th1 phenotype. These immune cells are central to the immune regulation and inflammatory responses in RA [55]. Thus, the role of CRP extends beyond merely being a marker of inflammation; it actively participates in the immune and inflammatory responses induced by smoking. Past research indicates that CRP in RA not only serves as a biomarker of systemic inflammation but also acts as an immunoregulatory factor involved in inflammation pathways related to RA [56]. Higher CRP levels are associated with increased risks of various comorbidities in RA patients, including cardiovascular diseases, diabetes, metabolic syndrome, pulmonary diseases, and depression. Moreover, managing RA can reduce CRP levels, potentially positively impacting the incidence and severity of these comorbidities [26]. In RA patients without a history of cardiovascular events, CRP levels correlate closely with the risk of future cardiovascular events over the next ten years. Research shows that for every 20 mg/L increase in CRP levels, there is a 1% increase in the predicted ten-year cardiovascular risk, according to the ERS-RA score. This suggests that actively managing residual inflammation in RA patients may further reduce the incidence of cardiovascular events [57]. These insights reinforce our understanding of the mechanisms by which smoking influences RA development through inflammatory pathways and indicate that monitoring and managing CRP levels could be valuable in preventing and treating smoking-related RA. Targeting CRP and the associated inflammatory pathways could alleviate the RA symptoms caused by smoking and improve clinical outcomes for patients. Emphasizing this in public health strategies, by reducing smoking exposure and raising awareness of early diagnosis and treatment of RA, could effectively reduce the social and economic burden of RA.

To explore the potential mechanisms linking smoking to the development of RA, this study focused on SNPs significantly associated with both conditions and conducted GO and KEGG enrichment analyses. These analyses aimed to reveal the potential roles of these genes in RA and their involvement in biological pathways. The results indicated a predominant enrichment of genes associated with smoking and RA in neural system pathways, especially in the axon guidance pathway. This suggests that smoking may influence RA not only through direct inflammatory pathways but also by altering the structure and function of the nervous system, thereby contributing to RA development. The axon guidance pathway is crucial for neuronal extension and positioning during embryogenesis [58,59]. Increasing evidence supports the role of axon guidance molecules in inflammation. For example, Netrin-1 can inhibit leukocyte migration and decrease pro-inflammatory cytokine production [60]. Similarly, molecules such as Semaphorin3A are implicated in the pathology of various chronic inflammatory diseases by modulating immune cell function and the angiogenic factors involved in inflammation [61]. Furthermore, recent research indicates that measuring Peptidoglycan Recognition Protein 1 (PGLYRP-1), Netrin-1, and miR-142-3p together can provide prognostic value for RA patients [62]. Notably, the expression of the axon guidance molecule Sema3A is significantly elevated in RA patients, positively correlating with inflammatory markers and being associated with autoantibody production and bone destruction [63]. Previous studies, including interaction analyses of gene and pathway data from GWAS, have identified significant interactions between the axon guidance signaling pathway and smoking behaviors. These interactions may play a crucial role in the etiology of diseases such as pancreatic cancer [64]. Additionally, this study observed significant enrichment of smoking-related genes in synaptic organization and neuronal cell body regions, suggesting that smoking may alter neuronal interactions and functions, thereby impacting immune cell behavior and the local inflammatory microenvironment, which are critical factors in RA pathogenesis. It is important to note that the specific mechanisms through which smoking-related neurobiological pathways influence RA remain unclear and warrant further experimental investigation.

This study did not find a statistically significant causal relationship between exposure to PM 2.5 μm and the risk of developing RA. This non-significant result should not be interpreted as evidence of no effect. Rather, it highlights potential limitations in the precision of exposure assessment or the complex nature of the interaction between PM 2.5 μm and genetic factors predisposing to RA. It underscores the necessity for ongoing research employing more refined and precise measurements of PM 2.5 μm exposure. Future studies could benefit from utilizing personal exposure monitoring or more detailed environmental modeling to capture individual exposure levels more accurately. Additionally, exploring interactions with other genetic and environmental factors could provide a more comprehensive understanding of the role of air pollution in RA pathogenesis.

While this study provides new insights into the relationship between environmental exposure and RA, it is not without limitations. Firstly, the data primarily come from European populations. This focus helps reduce biases due to population stratification but limits the broader applicability of the findings. The results may not fully apply to populations with different genetic backgrounds, making cross-ethnic studies essential to verify the universality and relevance of these findings across diverse genetic contexts. Secondly, the MR analysis uses genetic instrumental variables to represent exposure factors. Due to the current sample size and the limitations inherent in the additive regression model used, only a small proportion of the variance in exposure factors is explained. This limitation makes it difficult to detect subtle causal effects between complex traits. Additionally, although we attempted to explore the roles of iron metabolism and urinary biomarkers in the smoking–RA relationship through mediation analysis, the selection of these biomarkers was not exhaustive. They may not encompass all relevant physiological pathways, suggesting the possibility of other, unidentified mediating factors that could be influencing the relationship between smoking and RA. Lastly, the effect sizes derived from genetic correlation analysis are only estimates based on the current dataset and model and should not be equated with or replace effect sizes obtained from observational clinical studies. A more robust understanding and practical clinical insights can only be achieved by integrating genetic correlation analysis with traditional epidemiological studies, real-world research, bibliometric reviews, or meta-analyses. This comprehensive approach can provide a stronger evidence base that is beneficial for clinical practice.

## 5. Conclusions

This study utilized MR to explore the genetic connections and causal relationships between environmental exposures, with a focus on smoking and RA. Our findings indicate that lifelong smoking markedly elevates the risk for RA, particularly SPRA. Furthermore, CRP, serving as an inflammatory biomarker, acts as a mediator in the linkage between smoking and both RA and SPRA. This elucidates the potential mechanisms through which smoking exacerbates RA via inflammatory pathways. These insights not only shed light on the genetic and molecular pathways through which smoking intensifies RA but also highlight the critical need to reduce smoking exposure in public health initiatives. Additionally, these results provide a robust scientific foundation for future preventive and therapeutic strategies, deepen our understanding of RA’s complex etiology, and contribute to reducing the social and economic impacts of RA.

## Figures and Tables

**Table 1:** Genetic correlation between environmental exposures and RA or SPRA.

Trait1	Trait2	Genetic Correlation	SE	*p*-Value
Lifetime smoking	RA	0.1581	0.0361	1.22 × 10^−5^
Smoking initiation	RA	0.2106	0.0533	7.82 × 10^−5^
PM 2.5 μm	RA	0.0177	0.0681	0.7954
PM 2.5–10 μm	RA	−0.0029	0.1722	0.9865
PM 10 μm	RA	−0.0528	0.0691	0.4445
Nitrogen oxides	RA	0.0218	0.0667	0.7443
Nitrogen dioxide	RA	−0.0980	0.0623	0.1157
Lifetime smoking	SPRA	0.1039	0.0377	0.0058
Smoking initiation	SPRA	0.1454	0.0558	0.0092
PM 2.5 μm	SPRA	0.0746	0.0659	0.2573
PM 2.5–10 μm	SPRA	0.1331	0.1785	0.4559
PM 10 μm	SPRA	0.0169	0.0747	0.8213
Nitrogen oxides	SPRA	0.0695	0.0653	0.2877
Nitrogen dioxide	SPRA	−0.0066	0.0642	0.9176

## Data Availability

The datasets used and analyzed during the current study are available from the corresponding author on reasonable request.

## Supplementary Materials

The following supporting information can be downloaded at: https://www.mdpi.com/article/10.3390/biomedicines13020513/s1. Table S1: Results of the Cochran Q,MR-Egger-intercept and MR-PRESSO tests for detecting horizontal and directional pleiotropy and between SNP-heterogeneity in the Mendelian randomization; Table S2: Reverse MR analysis results between RA and environmental exposures; Table S3: The results of the causal impact of lifetime smoking on mediator factors; Table S4: The results of the causal impact of potential mediator factors on RA or SPRA; Table S5: The mediation proportion of mediator in the causal relationship between lifetime smoking and rheumatoid arthritis or seropositive rheumatoid arthritis; Table S6: The result of the conversion of SNPs into genes; Table S7: Top 12 clusters with their representative enriched terms in enrichment analysis. Supplementary STROBE checklist: STROBE-MR checklist of recommended items to address in reports of Mendelian randomization studies. References [65,66] are cited in the supplementary materials

## List of Abbreviations

RA, Rheumatoid arthritis; GWAS, genome-wide association studies; MR, Mendelian Randomization; LDSC, linkage disequilibrium score regression; SPRA, seropositive RA; SNRA, seronegative RA; PM, Particulate matter; NO_2_, nitrogen dioxide; NOx, nitrogen oxides; ACPA, anti-cyclic citrullinated peptide antibodies; RF, rheumatoid factor; LUR, land use regression; SNPs, Single Nucleotide Polymorphisms; WR, Wald ratio; IVW, inverse-variance weighted; GO, Gene Ontology; KEGG, Kyoto Encyclopedia of Genes and Genomes; BP, biological processes; MF, molecular functions; CC, cellular components; OR, odds ratios; CI, confidence intervals.
