# Supplementary material for: Causal Relationships Between Environmental Exposures, Iron Metabolism, Hematuria Markers, and Rheumatoid Arthritis: An Investigation Using Mendelian Randomization
Source: Biomedicines. 2025 Feb 19;13(2):513. doi: 10.3390/biomedicines13020513 (PMC11852645; doi:10.3390/biomedicines13020513)
Supplement: Supplementary file 1 [file biomedicines-13-00513-s001.zip › Supplementary STROBE checklist.pdf]

STROBE-MR checklist of recommended items to address in reports of Mendelian randomization studies[1 2]

Item No.	Section	Checklist item	Page No.	Relevant text from manuscript
1	**TITLE and ABSTRACT**	Indicate Mendelian randomization (MR) as the study's design in the title and/or the abstract if that is a main purpose of the study	1	Causal Relationships between Environmental Exposures, Iron Metabolism, Hematuria Markers, and Rheumatoid Arthritis: A Mendelian Randomization Investigation.
	**INTRODUCTION**			
2	**Background**	Explain the scientific background and rationale for the reported study. What is the exposure? Is a potential causal relationship between exposure and outcome plausible? Justify why MR is a helpful method to address the study question	2-3	Observational studies often struggle to adequately adjust for confounding factors across populations; thus, high-quality research designs are still required to validate causal relationships effectively. Additionally, lifestyle factors such as smoking have long been considered significant risk factors for RA exacerbating disease onset and severity; yet the underlying mechanisms of how smoking contributes to RA development remain unclear.
3	**Objectives**	State specific objectives clearly, including pre-specified causal hypotheses (if any). State that MR is a method that, under specific assumptions, intends to estimate causal effects	3	This study aims to investigate the genetic associations between environmental exposures—including but not limited to smoking initiation, lifetime smoking, and various forms of air pollution like PM2.5, PM2.5-10, PM10, NO2, and NOx—and RA, alongside potential mediation by iron metabolism and hematuria markers. By implementing forward MR methods, we estimated the causal effects of environmental exposures on RA with robust validation through sensitivity analyses and LDSC.
	**METHODS**			
4	**Study design and data sources**	Present key elements of the study design early in the article. Consider including a table listing sources of data for all phases of the study. For each data source contributing to the analysis, describe the following:		
	a)	Setting: Describe the study design and the underlying population, if possible. Describe the setting, locations, and relevant dates, including periods of recruitment, exposure, follow-up, and data collection, when available.	4	Our research utilized data accessible from public databases; participants in the GWASs were of European descent.
	b)	Participants: Give the eligibility criteria, and the sources and methods of selection of participants. Report the sample size, and whether any power or sample size calculations were carried out prior to the main analysis	4	Five GWAS datasets relating to air pollution were selected including PM2.5 um (n=423796), PM2.5-10 um (n=423796), PM10 um (n=455314), Nitrogen

			dioxide (n=456380), and Nitrogen oxides (n=456380); air pollution indices were measured using land use regression (LUR) models28. Specifically, data on smoking initiation came from GSCAN meta-Genome-Wide Association Study summary statistics which defined it as a binary variable contrasting never smokers against smokers who had smoked over 100 cigarettes in their lifetime29. The lifetime smoking index derived from UK Biobank factored various aspects of participants' smoking habits including severity and duration of smoking, onset and cessation, eventually combined with an estimated half-life constant reflecting exponential decline impacts on health outcomes posed at specific times—ultimately serving as a multifaceted evaluative measure for smoking30.
c)	Describe measurement, quality control and selection of genetic variants	4-5	The selection of Single Nucleotide Polymorphisms (SNPs) for our study was conducted via a systematic and stringent process delineated as follows: (1) To ensure robust statistical significance, a genome-wide significance threshold of p≤5×10-8 was employed. This threshold is conventionally adopted in GWAS to mitigate the possibility of false positives arising from multiple testing. (2) SNPs displaying a minor allele frequency below 0.05 were meticulously excluded from our analyses to maintain reliable genetic variance. (3) SNPs harboring mismatched allele pairs, such as C/T and C/A, were eradicated from consideration. (4) Palindromic variants with ambiguous strands, like C/G or A/T, were also excised from our dataset, a precaution vital for avoiding strand ambiguity which could introduce errors during analysis. (5) Genetic variants that successfully passed the aforementioned filters underwent additional processing in Plink. This software facilitated clustering using specific parameters to identify lead SNPs with settings configured at a window size of 10000 kb and an r2 less than 0.001, using LDlink to eliminate all potential confounding SNPs (https://ldlink.nih.gov/?tab=ldtrait).

		d)	For each exposure, outcome, and other relevant variables, describe methods of assessment and diagnostic criteria for diseases	4	RA (ICD-10 code M05, M06) data were derived from the FinnGen Consortium's GWAS summary dataset available online (https://r9.finngen.fi)31 encompassing 12,555 RA cases and 240,862 controls. It detailed data for Seropositive rheumatoid arthritis (SPRA, n=377272) as well as Seronegative rheumatoid arthritis (SNRA, n=288912) allowing for more thorough validation.
		e)	Provide details of ethics committee approval and participant informed consent, if relevant	4	Our research utilized data accessible from public databases; participants in the GWASs were of European descent.
5	**Assumptions**		Explicitly state the three core IV assumptions for the main analysis (relevance, independence and exclusion restriction) as well assumptions for any additional or sensitivity analysis	5	A bidirectional MR approach was implemented to rigorously investigate the causal relationships between environmental exposures and RA on a genetic scale. SNPs with low F-statistics (<10) were excluded from our analysis to dodge significant weak instrument bias. The Wald ratio (WR) method was utilized when only a single SNP was available, whereas the inverse-variance weighted (IVW) method served as the primary methodology for estimating causal relationships between exposure and outcome when two or more SNPs were available. Sensitivity analyses were enriched by employing four additional MR methods including MR-Egger regression, simple mode, weighted median, and weighted mode. Heterogeneity in the IVW estimates was assessed using Cochran's Q test. The MR Egger intercept was evaluated to ascertain pleiotropy. The MR-PRESSO framework, which encompasses global and outlier tests, was deployed to detect and rectify pleiotropy and potential outliers. After exclusion of pertinent outliers, causal estimates were recalculated, cementing a robust causal inference that considered potential biases and confounding factors inherent in the data. Further two-step MR analyses were pursued to explore whether iron metabolism and hematuria markers mediate the causal relationship from exposure to outcome.
6	**Statistical methods: main analysis**		Describe statistical methods and statistics used		

		a)	Describe how quantitative variables were handled in the analyses (i.e., scale, units, model)	6	The proportional mediation is derived based on the formula in which β represents the total effect obtained from preliminary analyses, whereas β1 and β2 denote the impact of exposure characteristics on mediator factors and the impact of said mediator factors on outcomes, respectively: Proportion = (β1×β2)/β.
		b)	Describe how genetic variants were handled in the analyses and, if applicable, how their weights were selected	6	The R software, version 4.3.1, has been employed for conducting MR and other analytical methods, utilizing the "TwoSampleMR" package. The results are expressed in terms of odds ratios (OR) along with their 95% confidence intervals (CI) per standard deviation increment.
		c)	Describe the MR estimator (e.g. two-stage least squares, Wald ratio) and related statistics. Detail the included covariates and, in case of two-sample MR, whether the same covariate set was used for adjustment in the two samples	5	A bidirectional MR approach was implemented to rigorously investigate the causal relationships between environmental exposures and RA on a genetic scale. SNPs with low F-statistics (<10) were excluded from our analysis to dodge significant weak instrument bias. The Wald ratio (WR) method was utilized when only a single SNP was available, whereas the inverse-variance weighted (IVW) method served as the primary methodology for estimating causal relationships between exposure and outcome when two or more SNPs were available.
		d)	Explain how missing data were addressed	5	SNPs featuring mismatched alleles were excluded. This includes alleles such as C/T and C/A. Palindromic variants with ambiguous strands, such as C/G or A/T, were also removed from the dataset.
		e)	If applicable, indicate how multiple testing was addressed		Not applicable
7	**Assessment of assumptions**		Describe any methods or prior knowledge used to assess the assumptions or justify their validity	5	The Wald ratio (WR) method was utilized when only a single SNP was available, whereas the inverse-variance weighted (IVW) method served as the primary methodology for estimating causal relationships between exposure and outcome when two or more SNPs were available.
8	**Sensitivity analyses and additional analyses**		Describe any sensitivity analyses or additional analyses performed (e.g. comparison of effect estimates from different approaches, independent replication, bias analytic techniques, validation of instruments, simulations)	5	Sensitivity analyses were enriched by employing four additional MR methods including MR-Egger regression, simple mode, weighted median, and weighted mode. Heterogeneity in the IVW estimates was assessed using Cochran’s Q test.

			The MR Egger intercept was evaluated to ascertain pleiotropy. The MR-PRESSO framework, which encompasses global and outlier tests, was deployed to detect and rectify pleiotropy and potential outliers. After exclusion of pertinent outliers, causal estimates were recalculated, cementing a robust causal inference that considered potential biases and confounding factors inherent in the data.
9	**Software and pre-registration**		
	a) Name statistical software and package(s), including version and settings used	6	MR and other analytical approaches were employed in R (version 4.3.1) utilizing the "TwoSampleMR" package.
	b) State whether the study protocol and details were pre-registered (as well as when and where)	-	. Not applicable
	**RESULTS**		
10	**Descriptive data**		
	a) Report the numbers of individuals at each stage of included studies and reasons for exclusion. Consider use of a flow diagram	6	Adhering to the criteria for the selection of IVs, we carefully selected 7, 144, 57, 22, 355, 119, and 85 SNPs significantly associated with smoking initiation, lifetime smoking, PM 2.5µm, PM 2.5-10µm, PM 10µm, nitrogen dioxide, and nitrogen oxides respectively across the genome.
	b) Report summary statistics for phenotypic exposure(s), outcome(s), and other relevant variables (e.g. means, SDs, proportions)	6	Adhering to the criteria for the selection of IVs, we carefully selected 7, 144, 57, 22, 355, 119, and 85 SNPs significantly associated with smoking initiation, lifetime smoking, PM 2.5µm, PM 2.5-10µm, PM 10µm, nitrogen dioxide, and nitrogen oxides respectively across the genome.
	c) If the data sources include meta-analyses of previous studies, provide the assessments of heterogeneity across these studies		Not applicable
	d) For two-sample MR:<br>i. Provide justification of the similarity of the genetic variant-exposure associations between the exposure and outcome samples<br>ii. Provide information on the number of individuals who overlap between the exposure and outcome studies	6	Among the environmental exposures assessed, the IVW approach revealed that genetically determined levels of lifetime smoking (per 1-SD increase) were associated with an 85% increase in the odds of developing RA (OR=1.85; 95% CI: 1.36-2.52; p=1.02×10-4).

11	**Main results**				
		a)	Report the associations between genetic variant and exposure, and between genetic variant and outcome, preferably on an interpretable scale	6	We carefully selected 7, 144, 57, 22, 355, 119, and 85 SNPs significantly associated with smoking initiation, lifetime smoking, PM 2.5µm, PM 2.5-10µm, PM 10µm, nitrogen dioxide, and nitrogen oxides respectively across the genome.
		b)	Report MR estimates of the relationship between exposure and outcome, and the measures of uncertainty from the MR analysis, on an interpretable scale, such as odds ratio or relative risk per SD difference	6	Among the environmental exposures assessed, the IVW approach revealed that genetically determined levels of lifetime smoking (per 1-SD increase) were associated with an 85% increase in the odds of developing RA (OR=1.85; 95% CI: 1.36-2.52; p=1.02×10-4).
		c)	If relevant, consider translating estimates of relative risk into absolute risk for a meaningful time period		Not applicable
		d)	Consider plots to visualize results (e.g. forest plot, scatterplot of associations between genetic variants and outcome versus between genetic variants and exposure)		Figure 2,3
12	**Assessment of assumptions**				
		a)	Report the assessment of the validity of the assumptions	6	This finding was consistent in the weighted median model analysis (OR=0.71; p=0.01). The remaining three MR analyses did not detect statistically significant associations but demonstrated similar trends of variation.
		b)	Report any additional statistics (e.g., assessments of heterogeneity across genetic variants, such as I^2, Q statistic or E-value)	6	Further sensitivity analysis for lifetime smoking is documented in Table S1. MR-Egger regression intercept analysis indicated no significant pleiotropy. Cochran's IVW Q-test suggested potential heterogeneity among instrumental variables; subsequent MR-PRESSO analysis identified outliers (rs12481282, rs62155874, rs732083), and upon correction for these outliers, the association remained significant (PRESSO Outliers corrected OR=2.06; p=2.18×10-6).
13	**Sensitivity analyses and additional analyses**				
		a)	Report any sensitivity analyses to assess the robustness of the main results to violations of the assumptions	6	Further sensitivity analysis for lifetime smoking is documented in Table S1. MR-Egger regression

				intercept analysis indicated no significant pleiotropy. Cochran's IVW Q-test suggested potential heterogeneity among instrumental variables; subsequent MR-PRESSO analysis identified outliers (rs12481282, rs62155874, rs732083), and upon correction for these outliers, the association remained significant (PRESSO Outliers corrected OR=2.06; p=2.18×10-6).
		b) Report results from other sensitivity analyses or additional analyses	6	Further sensitivity analysis for lifetime smoking is documented in Table S1. MR-Egger regression intercept analysis indicated no significant pleiotropy. Cochran's IVW Q-test suggested potential heterogeneity among instrumental variables; subsequent MR-PRESSO analysis identified outliers (rs12481282, rs62155874, rs732083), and upon correction for these outliers, the association remained significant (PRESSO Outliers corrected OR=2.06; p=2.18×10-6).
		c) Report any assessment of direction of causal relationship (e.g., bidirectional MR)	7	The results delineated that the genetic susceptibilities of RA, SPRA, and SNRA imparted no influence on any environmental exposures, as depicted in Table S2.
		d) When relevant, report and compare with estimates from non-MR analyses	8	GWAS summary data for environmental exposures, RA, and SPRA were utilized for a genetic correlation analysis. As illustrated in Table 1, consistent with MR study outcomes, there exists a significant positive genetic correlation between lifetime smoking and RA (correlation coefficient=0.158, p=1.22×10-5) and SPRA (correlation coefficient=0.104, p=5.81×10-3). Notably, despite the absence of a meaningful causal link revealed in MR analyses, LDSC analysis identified a significant positive genetic correlation between smoking initiation and both RA or SPRA.
		e) Consider additional plots to visualize results (e.g., leave-one-out analyses)	-	-
	**DISCUSSION**			
14	**Key results**	Summarize key results with reference to study objectives	8	By analyzing large-scale Genome-Wide Association Study (GWAS) data from European populations, we discovered that lifelong smoking significantly elevates the risk of developing RA, especially Seropositive RA (SPRA). Additionally, C-

					reactive protein (CRP), as an inflammatory biomarker, appears to mediate the relationship between smoking and both RA and SPRA.
15	**Limitations**		Discuss limitations of the study, taking into account the validity of the IV assumptions, other sources of potential bias, and imprecision. Discuss both direction and magnitude of any potential bias and any efforts to address them	11	While this study provides new insights into the relationship between environmental exposure and RA, it is not without limitations. Firstly, the data primarily comes from European and Asian populations. This focus helps reduce biases due to population stratification but limits the broader applicability of the findings. Results may not fully apply to populations with different genetic backgrounds, making cross-ethnic studies essential to verify the universality and relevance of these findings across diverse genetic contexts. Secondly, the MR analysis uses genetic instrumental variables to represent exposure factors. Due to the current sample size and the limitations inherent in the additive regression model used, only a small proportion of the variance in exposure factors is explained. This limitation makes it difficult to detect subtle causal effects between complex traits. Additionally, although we attempted to explore the roles of iron metabolism and urinary biomarkers in the smoking-RA relationship through mediation analysis, the selection of these biomarkers was not exhaustive. They may not encompass all relevant physiological pathways, suggesting the possibility of other, unidentified mediating factors that could be influencing the relationship between smoking and RA. Lastly, the effect sizes derived from genetic correlation analysis are only estimates based on the current dataset and model and should not be equated with or replace effect sizes obtained from observational clinical studies. A more robust understanding and practical clinical insights can only be achieved by integrating genetic correlation analysis with traditional epidemiological studies, real-world research, bibliometric reviews, or meta-analyses. This comprehensive approach can provide a stronger evidence base that is beneficial for clinical practice.
16	**Interpretation**				
		a)	Meaning: Give a cautious overall interpretation of results in the context of their limitations and in comparison with other studies	8	This study conclusively demonstrates a causal link between lifelong smoking and both RA and SPRA,

underscoring the direct impact of smoking behavior on increased RA risk. Our results show that smoking is not only associated with a generalized risk for RA but is particularly linked with the SPRA phenotype. These findings align with previous observational studies that frequently reported a connection between smoking and heightened RA risk[6]. However, by utilizing genetic instrumental variables, this study mitigates potential confounders and reverse causality, thus providing stronger evidence for smoking as a risk factor for RA. A prospective cohort study of 34,101 Swedish women aged 54 to 89 demonstrated significant correlations between the intensity and duration of smoking and the risk of developing RA, suggesting that even moderate smoking can elevate RA risk, which remains significant for up to 15 years post-cessation[7]. A large-scale prospective study based on the Nurses' Health Study (NHS) and NHSII examined the impact of smoking status, intensity, cumulative exposure, and duration since quitting on the development of RA and its serologic phenotypes in women[8]. The findings indicated that current smokers face a heightened risk for all types of RA, particularly SPRA, and that this risk diminishes progressively with time after quitting. Women who had quit smoking for more than 30 years exhibited a significantly reduced risk of developing SPRA compared to recent quitters, though the risk for RA was still marginally elevated even 30 years post-cessation. Another cohort study also demonstrated a positive correlation between smoking and SPRA, indicating that smoking increases the susceptibility to SPRA in individuals carrying the HLA-DRB1 shared epitope. In contrast, the relationship between smoking and Seronegative RA (SNRA) was either absent or minimal[9]. Unlike smoking, other respiratory irritants such as air pollution, organic dust, asbestos, and silica are linked not only to an increased risk of SPRA but also to SNRA. The mechanisms might involve pulmonary inflammation that generates new antigens promoting RA progression, alongside other potential pathways like cytokine production, T-cell polarization, epigenetic alterations, and local microbiome modulation[10]. This study conclusively demonstrates a causal link between lifelong

					smoking and both RA and SPRA, underscoring the direct impact of smoking behavior on increased RA risk. Our results show that smoking is not only associated with a generalized risk for RA but is particularly linked with the SPRA phenotype. These findings align with previous observational studies that frequently reported a connection between smoking and heightened RA risk[6]. However, by utilizing genetic instrumental variables, this study mitigates potential confounders and reverse causality, thus providing stronger evidence for smoking as a risk factor for RA.
		b)	Mechanism: Discuss underlying biological mechanisms that could drive a potential causal relationship between the investigated exposure and the outcome, and whether the gene-environment equivalence assumption is reasonable. Use causal language carefully, clarifying that IV estimates may provide causal effects only under certain assumptions	10-11	the study observed significant enrichment of smoking-related genes in synaptic organization and neuronal cell body regions, suggesting that smoking may alter neuronal interactions and functions, thereby impacting immune cell behavior and the local inflammatory microenvironment, which are critical factors in RA pathogenesis.
		c)	Clinical relevance: Discuss whether the results have clinical or public policy relevance, and to what extent they inform effect sizes of possible interventions	10	Additionally, C-reactive protein (CRP), as an inflammatory biomarker, appears to mediate the relationship between smoking and both RA and SPRA. This indicates that inflammatory pathways may be key mechanisms by which smoking influences RA progression. These insights provide a new perspective on how smoking exacerbates RA through genetic and molecular pathways and highlight the critical role of reducing smoking exposure in public health strategies.
17	**Generalizability**		Discuss the generalizability of the study results (a) to other populations, (b) across other exposure periods/timings, and (c) across other levels of exposure	11	the data primarily comes from European populations. This focus helps reduce biases due to population stratification but limits the broader applicability of the findings. Results may not fully apply to populations with different genetic backgrounds, making cross-ethnic studies essential to verify the universality and relevance of these findings across diverse genetic contexts.
	**OTHER INFORMATION**				
18	**Funding**		Describe sources of funding and the role of funders in the present study and, if applicable, sources of funding for the databases and original study or studies on which the present study is based	-	-

19	**Data and data sharing**	Provide the data used to perform all analyses or report where and how the data can be accessed, and reference these sources in the article. Provide the statistical code needed to reproduce the results in the article, or report whether the code is publicly accessible and if so, where	-	All the data are available if qualified authors apply for them.
20	**Conflicts of Interest**	All authors should declare all potential conflicts of interest	-	The authors declare that the research was conducted in the absence of any commercial or financial relationships that could be construed as a potential conflict of interest.

This checklist is copyrighted by the Equator Network under the Creative Commons Attribution 3.0 Unported (CC BY 3.0) license.

1. Skrivankova VW, Richmond RC, Woolf BAR, Yarmolinsky J, Davies NM, Swanson SA, et al. Strengthening the Reporting of Observational Studies in Epidemiology using Mendelian Randomization (STROBE-MR) Statement. JAMA. 2021;326,1614 – 1621.

2. Skrivankova VW, Richmond RC, Woolf BAR, Davies NM, Swanson SA, VanderWeele TJ, et al. Strengthening the Reporting of Observational Studies in Epidemiology using Mendelian Randomisation (STROBE-MR): Explanation and Elaboration. BMJ. 2021;375:n2233.
